# Factors Driving Bacterial Microbiota of Eggs from Commercial Hatcheries of European Seabass and Gilthead Seabream

**DOI:** 10.3390/microorganisms9112275

**Published:** 2021-11-01

**Authors:** Babak Najafpour, Patricia I. S. Pinto, Katerina A. Moutou, Adelino V. M. Canario, Deborah M. Power

**Affiliations:** 1 Centro de Ciências do Mar (CCMAR/CIIMAR), Universidade do Algarve, 8005-139 Faro, Portugal; bnajafpour@ualg.pt (B.N.); ppinto@ualg.pt (P.I.S.P.); acanario@ualg.pt (A.V.M.C.); 2 Department of Biochemistry and Biotechnology, University of Thessaly, Biopolis, 41221 Larissa, Greece; kmoutou@bio.uth.gr

**Keywords:** microbiome, broodstock water, disinfection, hatchery, teleost eggs

## Abstract

A comprehensive understanding of how bacterial community abundance changes in fishes during their lifecycle and the role of the microbiota on health and production is still lacking. From this perspective, the egg bacterial communities of two commercially farmed species, the European seabass (*Dicentrarchus labrax*) and the gilthead seabream (*Sparus aurata*), from different aquaculture sites were compared, and the potential effect of broodstock water microbiota and disinfectants on the egg microbiota was evaluated. Moreover, 16S ribosomal RNA gene sequencing was used to profile the bacterial communities of the eggs and broodstock water from three commercial hatcheries. Proteobacteria were the most common and dominant phyla across the samples (49.7% on average). *Vibrio* sp. was the most highly represented genus (7.1%), followed by *Glaciecola* (4.8%), *Pseudoalteromonas* (4.4%), and *Colwellia* (4.2%), in eggs and water across the sites. Routinely used iodine-based disinfectants slightly reduced the eggs’ bacterial load but did not significantly change their composition. Site, species, and type of sample (eggs or water) drove the microbial community structure and influenced microbiome functional profiles. The egg and seawater microbiome composition differed in abundance but shared similar functional profiles. The strong impact of site and species on egg bacterial communities indicates that disease management needs to be site-specific and highlights the need for species- and site-specific optimization of disinfection protocols.

## 1. Introduction

Knowledge about the symbiotic interdependency between complex multicellular eukaryotes and their microbiota is changing our understanding of animal biology [1,2]. Studies of the microbiota in humans have highlighted the complexity and unexpected role that the microbiome plays in development and physiology [3,4]. This has transformed our understanding of the importance of microbiota for health and disease and opened up a new research frontier [5]. In terrestrial animal production systems, the role of the microbiota in traits of interest and as a means to control pathogens and deliver alternatives to conventional pharmaceuticals has stimulated high interest [6]. Benefits already accrued are linked to host nutrition, the promotion of epithelial barrier function, stimulation of the immune system, and protection against colonization by pathogens [7]. The recent massive increase in knowledge about the composition and function of microbes, including non-culturable bacteria in a wide range of ecological niches, has been made possible by 16S ribosomal RNA (rRNA) gene amplicon sequencing and meta-transcriptomics [8,9].

The high number and diversity of bacteria and viruses in aquatic systems mean that aquatic organisms such as fish are exposed to a larger number and diversity of microorganisms than terrestrial vertebrates, and this is proposed to have influenced their physiology [10]. This suggests that improved knowledge about fish microbiota may create new opportunities for the management of aquaculture production. This notion has accelerated microbiome research, particularly in species of commercial interest for aquaculture [11]. Most studies so far have focused on the role of the microbiota in specific tissues, mostly the gut of adult fish, due to the recognized importance of this tissue in digestion and the assimilation of essential nutrients and in contributing to good growth and health [12,13,14]. Furthermore, considerable interest exists in the potential benefits that may be obtained by manipulating the microbiota of the fish gut using probiotics and prebiotics in the diet [15,16,17]. Such studies have revealed the potential role of the gut microbiota in resistance to pathogens, improved growth, lipid metabolism, and the immune response [18]. 

Studies investigating fish egg- and embryo-associated microbiota have so far mainly targeted potential pathogens such as *Leucothrix mucor*, *Flexibacter ovolyticus*, *Flavobacterium columnare*, and *Pseudoalteromonas piscicida* [19,20,21]. Although the use of metagenomics to determine the global community of microbes, or microbiota, of fish eggs and embryos is still at an early stage, a positive impact of the intrinsic microbiota on fish egg quality has been recognized [22]. Dysbiosis has been linked to the colonization of eggs by pathogenic bacteria, leading to high mortality during incubation, and it is now clear that the fish egg microbiota shapes the larval microbiota [11,20] and may influence subsequent larval performance [23]. Recent studies of eggs from channel catfish (*Ictalurus punctatus*) [24] and ballan wrasse (*Labrus bergylta*) [25] suggest that their microbiota may be influenced by the holding tank, genetics (family effects), or egg disinfection procedures. Clearly, a better understanding of the factors influencing the egg microbiota of aquaculture species can contribute information relevant for improving management and may help to identify whether the microbiota has a role in determining egg quality. 

The European seabass (*Dicentrarchus labrax*) and the gilthead seabream (*Sparus aurata*) are the most important farmed fish species in the Mediterranean region, with a combined production of 407,673 tonnes in 2019, with the highest contribution from Turkey [26]. The hatchery stage of production is crucial for industry sustainability. Identification of the factors that lead to unpredictable production quality due to high mortality rates or poor-quality eggs and subsequent developmental stages is a priority for gilthead seabream and European seabass hatcheries [11,27]. 

The main objectives of the present metagenomic study, in the context of the hatchery production of gilthead seabream and European seabass, were therefore to (i) establish the composition and diversity of the bacterial community in eggs collected from three different industrial sites, (ii) assess the impact of disinfection protocols on the egg microbiome, and (iii) evaluate the contribution of the microbiota in the broodstock tank water to the egg bacterial community. This is, to our knowledge, the first metagenomic study of the fish egg microbiome and its modulation by environmental variables in different sites across the Mediterranean coastal waters of Greece.

## 2. Materials and Methods

### 2.1. Broodstock Culture Conditions and Disinfection Protocol

**Figure 1:**
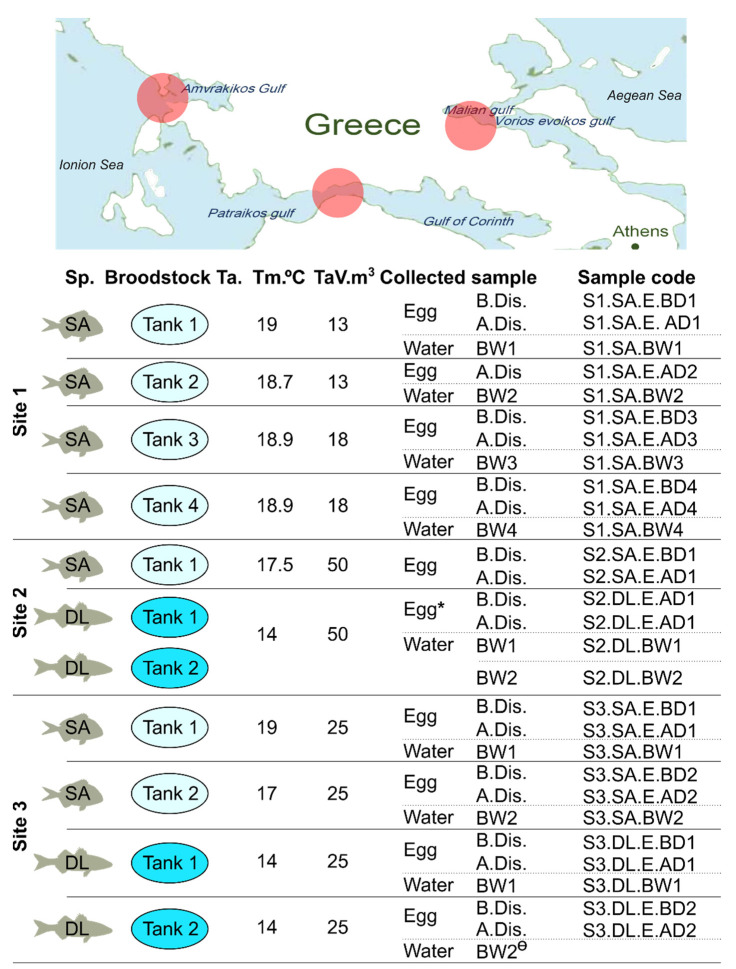
A schematic representation of the approximate location of the hatcheries and the experimental design. Egg and water samples were collected from the three different sites/hatcheries (red circles on the map) and different broodstock in January 2020. Egg samples of the gilthead seabream (SA) and the European seabass (DL) were taken before (B.Dis.) and after (A.Dis.) standard egg disinfection procedures at each site. For easy tracking of samples and their results, sample codes were assigned to each of the collected samples (see last column). Sp. (species); BW (broodstock water); Ta. (tank); Tm. (temperature in °C); TaV. (tank volume in m^3^). Note: * the eggs from two broodstock were mixed in this case. Ө—sequencing of this specific water sample did not proceed because insufficient DNA was obtained during extraction.

Eggs from 11 different broodstock (BS) tanks from European seabass and gilthead seabream were obtained from three hatcheries located at different sites in Greek coastal waters (Figure 1 and Table 1) in January 2020. The sites were designated 1 to 3 and samples designated according to their site origin, but due to the potential commercial sensitivity of the information, samples and site origin are not linked. The different gilthead seabream and European seabass BS from the same sites were maintained in separate sea water flow through tanks with independent water supplies. The experimental design, experimental tank temperatures and volumes, and sample references are provided in Figure 1. 

Different protocols were used for egg disinfection at the different sites; these were the standard operating protocols established for routine use in each of the commercial hatcheries that collaborated in the study. In site 1, gilthead seabream eggs (≅1 kg) were immersed in 10 L of seawater containing 0.25 mL GERM-iod (18 mg iodine/mL, final concentration 0.45 mg iodine/L) for 3 min. Eggs were rinsed after treatment with clean seawater and stocked directly into larval tanks. In site 2, gilthead seabream eggs (≅1 kg) were immersed in 10 L of seawater containing 50 mL of Ovadine (Syndel, 10% polyvinyl pyrrolidine iodine with 1% available iodine) for 7 min. The eggs were not rinsed and were directly stocked into the incubator tanks (500 L). European seabass eggs (≅1 kg) followed a similar protocol to gilthead seabream egg treatments, with the exception that 70 mL of Ovadine was used and the egg incubation tanks were of 200 L. In site 3, gilthead seabream and European seabass eggs were immersed in 10 L of seawater containing 35 mL of Buffodine (Evans Vanodine International plc; iodine-based and neutral pH disinfectant) for 5 min. After treatment, the eggs were washed in clean seawater and stocked into larval tanks (1000 L).

### 2.2. Sample Collection

European seabass and gilthead seabream eggs and broodstock water samples (BW) were collected from each of the three hatchery sites. Floating eggs were collected into a sterile beaker by scooping them from the surface of the water into an egg collector. The eggs were recovered from the seawater by gently straining through a tea strainer, and rinsed in sterile seawater before transfer to sterile 50 mL tubes containing RNA later (Sigma-Aldrich, Madrid, Spain; eggs:RNA later *v*/*v* 1:10). Nineteen egg samples were collected, 9 before and 10 after disinfection. A total of 10 BW samples (400 mL each) were collected by scooping seawater directly from the broodstock tank using a sterile beaker and transferred into 1 L bottles containing 50 mL DESS solution (0.25 M disodium EDTA, pH 8.0, 20% dimethyl sulfoxide, saturated with sodium chloride [28]), mixed well and stored at 4 °C. 

### 2.3. DNA Extraction

Total DNA from the 29 samples (19 egg samples and 10 water samples) was extracted using a DNeasy Blood & Tissue Kit (Qiagen, Hamburg, Germany) following the manufacturer’s instructions, modified to include pre-digestion with lysozyme and RNAse treatment [29]. The optimization of the initial mechanical disruption step was established for each sample type, as was the weight of eggs, water volume, lysozyme concentration, and buffers used in the present study. 

For most egg samples, 30 mgs of eggs suspended in RNA later was used for disruption and yielded sufficient DNA for 16S rRNA library construction. For some samples (S1.SA.E.AD3, S1.SA.E.AD4, S2.SA.E.BD1, S2.SA.E.AD1, S3.DL.E.AD2, S3.DL.E.BD1, and S3.DL.E.AD1), 90 mg of eggs was extracted to ensure sufficient DNA yield for subsequent analysis. Lysis mix (200 µL of lysis buffer 20 Mm Tris-HCl, pH 8; 2 mM sodium EDTA; 1.2% Triton X-100; 40 mg/mL lysozyme mixed with 200 µL of AL buffer from the Qiagen kit) was added to each egg sample with two iron beads (Qiagen stainless steel beads of 5 mm) per sample. Initial mechanical disruption of the eggs was carried out using 3 cycles of 30 s at 30 Hz in a Tissue Lyser (Qiagen). The iron beads were then removed and 400 mg of 0.1 mm zirconia/silica beads per tube was added and a second step of mechanical disruption (3 cycles of 5 min at 25 Hz) targeting the bacterial cells was performed.

For BW (400 mL), the particulate matter containing the microorganisms was concentrated into a single 50 mL sterile tube by centrifugation of the water at 16,100 *g* for 20 min at 4 °C. The resulting pellet was suspended at room temperature in 2 mL sterile extraction tubes (Sarstedt, Nümbrecht, Germany) by adding 400 µL of the lysis mix and approximately 400 mg of 0.1 mm zirconia/silica beads (Biospec). Mechanical disruption of the pellet was carried out at room temperature in the Tissue Lyser using 3 cycles of 5 min at 25 Hz.

### 2.4. 16S rRNA Library Construction and Sequencing

The extracted DNA was shipped on ice to Stab Vida, Lda (Lisbon, Portugal), where the integrity and quantity of the DNA was confirmed using 1.5% agarose gel electrophoresis and a Qubit 2 fluorometer (ThermoFisher Scientific, Lisbon, Portugal). Metagenomic amplicon library construction was carried out using an Illumina 16S Metagenomic Sequencing Library preparation protocol, with 12.5 ng DNA per sample, and primers targeting the V3 and V4 hypervariable regions of the 16S rRNA gene for amplification [30]. From the 29 samples, 28 libraries were generated and successfully sequenced by Stab Vida, Lda using a MiSeq Reagent Kit v3 and generated 300 bp paired-end sequencing reads in an Illumina MiSeq instrument.

### 2.5. Sequence Processing and Bioinformatics 

The quality of the raw sequencing reads was evaluated using FastQC [31], and the reads were denoised using the DADA2 plugin of QIIME 2 v2020.2 [32,33] and included read filtering, dereplication, and chimera filtering. After preliminary analysis to evaluate the presence of host DNA in the data generated, an additional filtering step was introduced. Specifically, the operational taxonomic units (OTUs) generated were queried using BLAST against an in-house sequence database, created using the two host DNA genomes, gilthead seabream *Sparus aurata* (Genbank assembly accession GCA_900880675) and European seabass *Dicentrarchus labrax* (GCA_000689215). The parameter settings for host-specific DNA filtering were: word size 11, match point 2, mismatch point-3, gap existence-5/gap extension-2. OTUs corresponding to contaminating host DNA were removed using the QIIME 2 filtering options, and the remaining OTUs were analyzed. 

The rarefaction curves for the samples of each site were plotted using the *rarecurve* function in the R package vegan v 2.5-6. QIIME 2 v2020.2 was used for the identification and classification of OTUs using the scikit-learn classifier against the SILVA (release 132 QIIME) database [34], with a cut-off threshold set at 97% similarity. For classification purposes, only OTUs in the dataset containing at least 10 sequence reads were considered. QIIME 2 was also used to calculate commonly used alpha- and beta-diversity metrics (Shannon’s diversity index and Bray–Curtis distance) and the output was imported into R to produce principal coordinate analysis (PCoA) and for visualization of the data using the packages qiime2R v 0.99.6 and ggplot2 v 3.3.5.

### 2.6. Functional Analysis

Functional predictions based on the 16S rRNA metagenomics profiles were run on the web-based platform Microbiome Analyst [35] using the Tax4Fun method [9]. The associations between functional categories (Kyoto Encyclopedia of Genes and Genomes, KEGG pathways) and the experimental factors (site, disinfection, species, and sample type) were tested using the global test algorithm [36]. 

### 2.7. Quantitative Analysis of 16S rRNA Gene 

The 16S rRNA gene was quantified in genomic extracts, in duplicate reactions, by quantitative polymerase chain reaction (qPCR), run in a Bio-Rad CFX96 qPCR Instrument (Bio-Rad Laboratories, Hercules, CA, USA). The effects of aquaculture site, species, and disinfection on the bacterial load of the eggs were evaluated using the quantified 16S rRNA gene across samples. The primers used for estimation of bacterial loads were those recommended by the Earth Microbiome Project (http://www.earthmicrobiome.org/protocols-and-standards/16s/ (accessed on 15 June 2020)) and targeted a fragment of approx. 300 bp between positions 515 and 806 of the 16S rRNA gene. The sense primer 16S-515fbY or 515F (Parada) sequence is 5′-GTGYCAGCMGCCGCGGTAA-3′ and the antisense primer 16S-806rbN or 806R(Apprill) is 5′-GGACTACNVGGGTWTCTAAT-3′ [37,38,39], using standard codes for degenerate bases (Y = C or T; N = A, C, T, or G; M = A or C; W = A or T; V = A, C, or G). 

The final qPCR reaction volume was 10 µL and contained 200 nM of each primer, 2 µL of the template cDNA (10 ng), and 5 µL of 2× Forget-Me-Not™ EvaGreen^®^ qPCR Master Mix (Biotium). Thermocycling conditions were 95 °C for 2 min, followed by 40 cycles of 95 °C for 5 s, 50 °C for 10 s, and 72 °C for 10 s, with a final melting curve generated by increasing the temperature from 60 °C to 95 °C, with increments of 0.5 °C each 10 s. The absence of non-specific amplification and primer dimers was verified by analysis of melting curves and running representative amplification products on 2% agarose gels, which confirmed single peaks and products of the expected size. Standard curves were included in all qPCR plates and were prepared from serial dilutions of a plasmid containing a 1013 bp fragment of the 16S rRNA gene from *Mycoplasma* cloned and sequenced from a *Solea senegalensis* gut sample [29]. Control reactions were added to all qPCR plates, including a no template control to confirm the absence of reagent contamination. The reaction efficiency of the qPCR and coefficient of determination (r^2^) were 95.6% and 0.986, respectively.

### 2.8. Statistics 

Overall, four variables were considered in the statistical tests to analyze the bacterial communities: (i) disinfection (eggs before versus eggs after disinfection), (ii) sample type (broodstock water vs. eggs), (iii) species (gilthead seabream vs. European seabass), and (iv) geographical location of the aquaculture site (site 1 vs. site 2 vs. site 3). Statistical significance in all tests was set at *p* < 0.05. Data normality and homogeneity were tested using the Shapiro–Wilk normality test. Statistical analyses were performed in the R environment.

To compare alpha-diversity by Shannon indexes for each of the 4 variables, one-way ANOVA (analysis of variance) was applied. To evaluate beta-diversity, data homogeneity was controlled using the *betadisper* function (evaluating beta-dispersion) and the *permutest* function. Based on the principal coordinate analysis (PcoA) of beta-diversity data, permutational analysis of variance (PERMANOVA) using the *adonis* function and Bray–Curtis distances were applied to test whether the overall microbial community differed with each variable under analysis. Differential abundance analyses were run using the R package ALDEx2 (1.20.0) to find features and pathways that had different abundance across variables. The centered log-ratio-transformed values were analyzed using a general linear model (glm) and Kruskal–Wallis tests. To specify features/OTUs with a significantly different relative abundance for the defined variables, the output was filtered using glm.ep based on the confidence interval of 95% (*p* < 0.05). 

The paired samples Wilcoxon test was used to compare qPCR 16S rRNA abundance measurements before and after disinfection and the Kruskal–Wallis test to compare abundance between species and site. 

## 3. Results

### 3.1. Sequencing and Rarefaction Outcome

A total of 11.7 million paired-end reads (with an average read number of 417,809 ± SD of 158,260) were produced from the 28 metagenomic 16S libraries. Sequence assembly yielded 3 million paired-reads, with a mean of 106,893 reads per library, which, after quality control and trimming of low-quality sequences, resulted in 2 million reads, with a mean of 72,072 sequences per library (Supplementary Table S1). Taxonomic classification of these reads identified a total of 1,819 unique features (OTUs).

**Figure 2:**
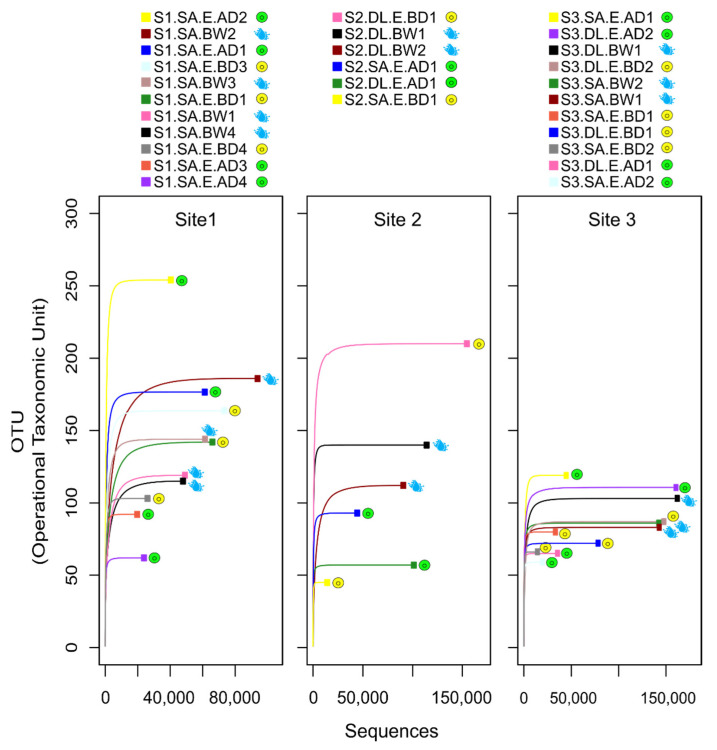
Rarefaction plots of the sequencing data from the 16S rRNA microbiome libraries. The rarefaction curves related to the eggs and water samples of each site (Site 1, Site 2, Site 3) are plotted separately. Symbols show different types of samples: eggs before disinfection = yellow circle; eggs after disinfection = green circle; BS water = blue drop. The sequences generated from each of the 28 libraries reached a plateau for all the samples, revealing that all bacterial diversity was covered. The rarefaction curves were plotted using the *rarecurve* function in the R package vegan (v 2.5-6). Information about the sample labels is presented in the legend of Figure 1.

The alpha rarefaction curves confirmed that the sequencing depth was sufficient to cover the microbial community diversity across samples, as they reached a plateau in all 28 libraries (Figure 2). Overall, less variation in OTUs between libraries was observed for site 3 compared to the other sites, and no obvious pattern was observed in the total number of OTUs in eggs before and after disinfection in any of the sites. However, in sites 1 and 3, the highest numbers of OTUs were in two specific egg samples after disinfection. In site 2, the highest number of OTUs was detected in one egg sample before disinfection.

### 3.2. Bacterial Community Taxonomic Composition

**Figure 3:**
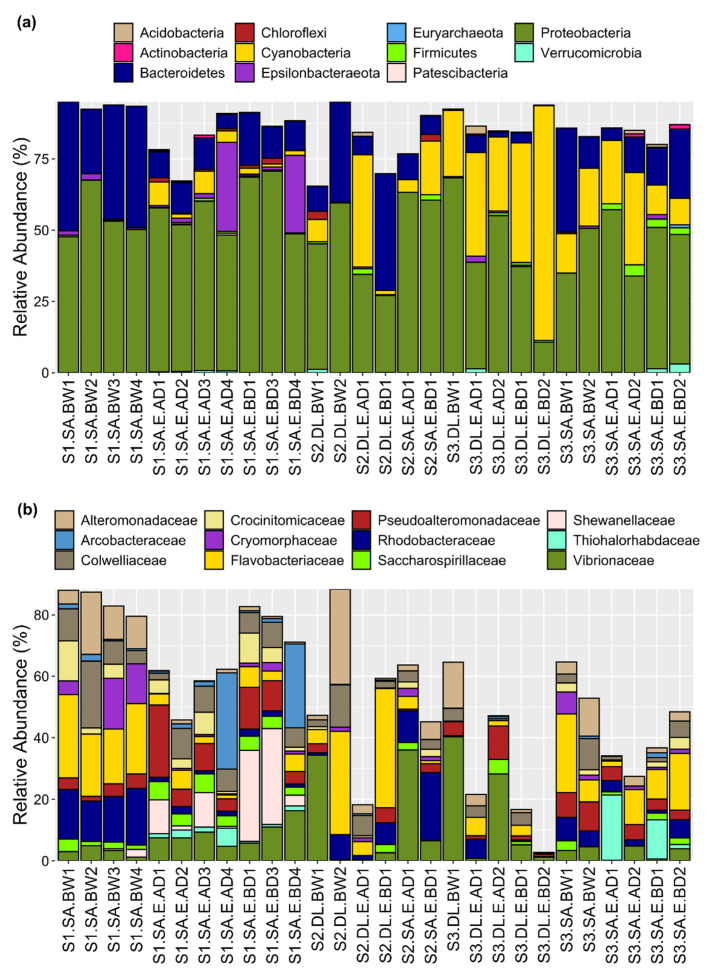
The relative abundance of the 11 phyla (**a**) and 12 families (**b**) across egg and water samples. The relative proportions of phyla and families (in % in relation to the total profiled microbiome) were plotted using the ggplot2 R package. Information about the sample labels is presented in the legend of Figure 1.

The bacterial composition and their relative abundance were determined in each sample at different taxonomic levels and the data are presented for all taxa detected at >1% in abundance across all samples (Supplementary Table S2). Proteobacteria were the dominant phylum and were highly represented in all the samples (mean = 49.7%, range = 10.7–70.7%) (Figure 3a). Bacteroidetes was the second most dominant phylum across samples (mean = 15.9, range = 0.11–45.2%) (Figure 3a). The highest relative abundance and representation of Cyanobacteria was observed in site 3 (mean = 28.9%, range = 9.3–42.0%), followed by site 2 (mean = 12, range = 0.01–39.4%) and site 1 (mean = 2.4, range = 0–8.2%) (Figure 3a). 

The two most abundant families that were represented in almost all samples irrespective of site or fish species were Flavobacteriaceae (mean = 10.3%, range = 0–38.7%) and Vibrionaceae (mean = 8.9%, range = 0.12–40.3%). Colwelliaceae, Rhodobacteraceae, Pseudoalteromonadaceae, and Alteromonadaceae (mean ≅ 5%) were the next most abundant families across all egg and water samples (Figure 3b). All families, except for Pseudoalteromonadaceae, were more abundant in water than in eggs (Figure 3b). Shewanellaceae (mean = 3.3%, range = 0–31.1%) showed high relative abundance in gilthead seabream egg samples from site 1 (Figure 3b).

**Figure 4:**
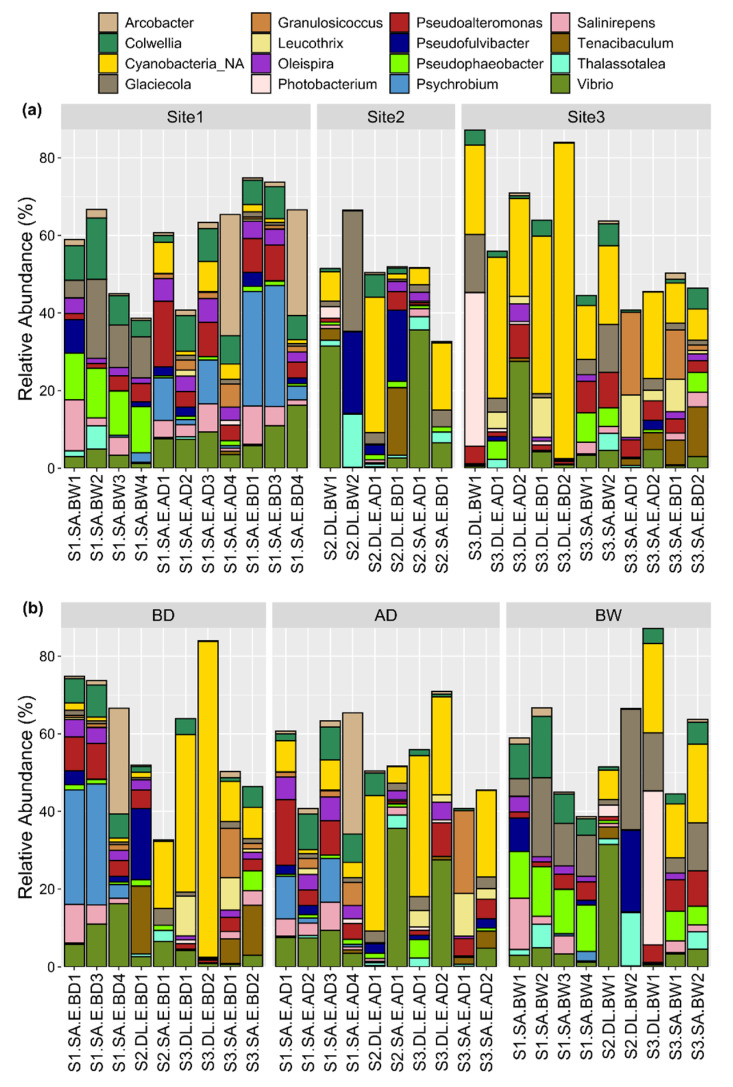
The relative percentage of the most abundant bacterial genera (top 15) across all egg and water samples. The bacterial genera present at the highest relative percentage, based on the sum of the percentage detected in all samples, were selected for presentation. The same data are rearranged and presented (using the ggplot2 R package) in relation to (**a**) the aquaculture site or (**b**) the type of sample including eggs (before disinfection, BD, and after disinfection, AD) and water samples (broodstock water, BW).

The relative abundance (%) of microbial genera for each sample is described in Supplementary Table S3. The 15 most abundant genera per aquaculture site are shown in Figure 4a and per sample type (eggs before and after disinfection, and broodstock water) in Figure 4b. Some bacterial genera were not specified (mean = 22.1%, range = 1.1–83.6) because they were not represented in the SILVA database (NA in Supplementary Table S2). Cyanobacteria (mean = 13.2%, range = 0.02–82.6%) were among the highest proportion of the 22.1% unidentified bacterial genera (Figure 4). *Vibrio* was the most represented genus in eggs and broodstock water at the three sites (mean = 7.1%, range = 0–35.6%). *Glaciecola* (mean = 4.8%, range = 0–31.0%), *Pseudoalteromonas* (mean = 4.4%, range = 0–16.8%), and *Colwellia* (mean = 4.2%, 0.07–15.8%) were the genera with the next highest relative abundance across the samples (Figure 4). 

Consideration of site-specific relative abundance of bacterial genus revealed that *Psychrobium* was very abundant in some egg samples from site 1 (mean = 3.32%, range = 0–31.1% across all samples). Cyanobacteria were abundant in most of the egg and in all water samples from site 3 and in some egg samples from site 2 (Figure 4a). *Glaciecola* and *Pseudophaeobacter* (mean = 3%, range = 0–12.8% across all samples) contributed at a higher relative proportion to the microbiota in water samples compared to egg samples (Figure 4b). Comparison of the bacterial community profiles before disinfection (BD) and after disinfection (AD) suggested that disinfection had a relatively mild effect on the microbiota overall, although some genera appeared to be more affected by disinfection. For example, *Psychrobium* abundance was substantially reduced in two gilthead seabream egg samples (S1.SA.E.AD1 and S1.SA.E.AD3) and *Pseudofulvibacter* was reduced in one European seabass egg sample (S2.DL.E.AD1) (Figure 4b). 

### 3.3. Bacterial Community Diversity

#### 3.3.1. Alpha-Diversity

**Figure 5:**
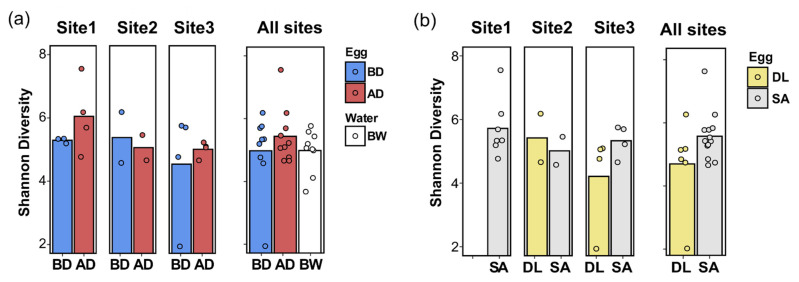
The Shannon index of alpha diversity. The Shannon index is presented according to (**a**) the sample type, including eggs (before disinfection, BD, and after disinfection, AD) and water samples (broodstock water, BW) across the three aquaculture sites (Site 1, Site 2, and Site 3), and (**b**) the species (gilthead seabream, SA, and European seabass, DL) across the three aquaculture sites (Site 1, Site 2, and Site 3), or in the representative plot on the right of each panel, compiling the average Shannon index for all three sites. No significant difference was observed in the Shannon index, by sample type, species, or site.

Shannon’s diversity indexes (reflecting the microbial community alpha-diversity) were not significantly modified by disinfection (egg before vs. egg after disinfectant usage), sample type (broodstock water vs. egg), species (gilthead seabream eggs vs. European seabass eggs), or site (site 1, site 2, or site 3) (Figure 5). 

#### 3.3.2. Beta-Diversity

**Figure 6:**
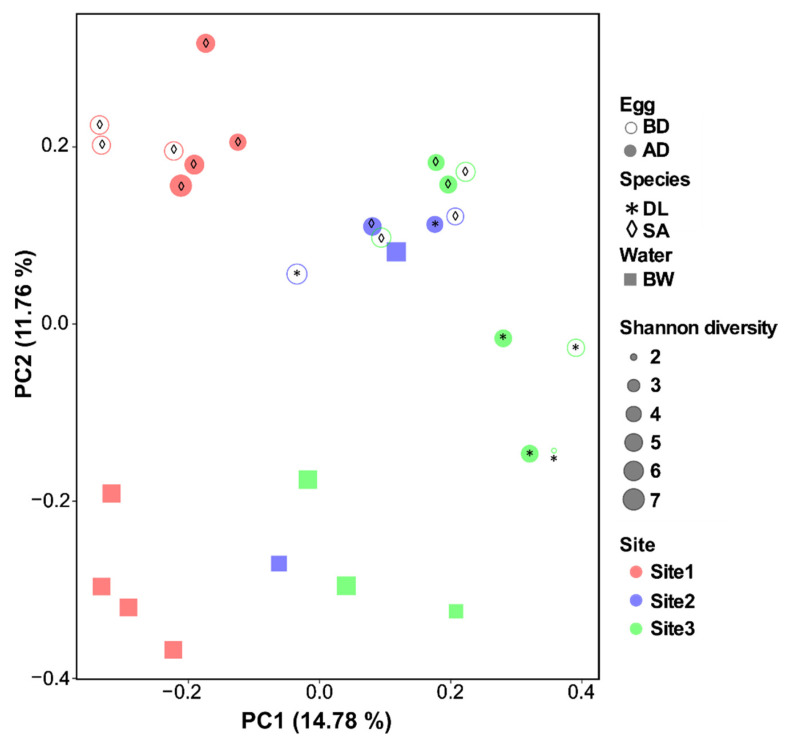
Visual representation of differences in the microbiota composition of eggs and water samples (beta diversity) using principal coordinates analysis (PcoA). Egg samples include eggs before (BD) and after (AD) disinfection from European seabass (DL, marked with * in the plot) and gilthead seabream (SA, marked with ◊ in the plot). Water samples were collected from broodstock tanks (BW) across three hatcheries/sites and also analyzed. PcoA analysis (Bray–Curtis distances) was run in the R environment using qiime2R and ggplot2 packages.

To visualize the differences between bacterial community composition and distance across all collected samples (eggs and water), PcoA analysis was performed and the plot displayed in a two-dimensional space (Figure 6). The distinction in the microbial communities between sample type (eggs vs. water) was most obvious in sites 1 and 3 compared to site 2 (Figure 6). No clear separation was observed in the bacterial composition of the eggs before and after disinfection (*p* = 0.96, Table 3). Site and species had a significant effect on the egg bacterial community, and the bacterial composition of the eggs and broodstock water was significantly different (Bray–Curtis distance analysis in PERMANOVA, *p* < 0.001, Table 2 and Table 3). 

### 3.4. Relative Abundance

There were significant differences in OTU relative abundance (*p* < 0.05) according to site, sample type, and species (Supplementary Table S4). Site comparisons of the bacterial abundance specified 73 OTUs with significantly different relative abundance (*p* < 0.05) and included 36 bacterial genera, such as *Oleispira*, *Colwellia*, *Psychrobium*, *Vibrio*, *Pseudoalteromonas*, *Psychromonas*, *Arcobacter* (listed in Supplementary Table S3, Site). Comparison of the egg microbial community with broodstock water identified 36 OTUs with significantly different relative abundance (*p* < 0.05) and included 14 genera, such as *Pseudomonas*, *Salinirepens*, *Colwellia*, *Psychrobium*, *Leucothrix*, *Pseudophaeobacter* (listed in Supplementary Table S4, Type). The relative abundance of 14 OTUs was significantly different in the comparison of gilthead seabream with European seabass eggs across the three sites (*p* < 0.05, listed in Supplementary Table S4, Species). *Pseudomonas* and *Photobacterium* were among the genera with higher relative abundance in European seabass eggs (*p* < 0.05), while *Vibrio* was more abundant in gilthead seabream eggs (*p* < 0.05). The genus of some OTUs with significant changes in their relative abundance was not identified in databases (represented by NA in Supplementary Table S4). Egg disinfection did not cause statistically significant changes in OTU abundance in any of the sites (*p* > 0.05).

### 3.5. Functional Prediction

In general, Tax4Fun functional predictions identified 6311 KEGG orthologous and 133 KEGG pathways (Supplementary Table S5). There was a significant association of 88 pathways with site, 7 pathways with disinfection, and 49 pathways with fish species (*p* < 0.05). No pathway was significantly associated with the type of sample (water vs. egg). The most abundant pathways were determined by mean of the relative abundance of the pathway across all samples (Table 4). Analysis of the “site” variable with the Kruskal–Wallis H test identified 10 significant pathways, based on the Benjamini–Hochberg-corrected *p*-value (BH): neomycin, kanamycin, and gentamicin biosynthesis (ko00524), fructose and mannose metabolism (ko00051), ascorbate and aldarate metabolism (ko00053), pentose and glucuronate interconversions (ko00040), tetracycline biosynthesis (ko00253), steroid hormone biosynthesis (ko00140), D-alanine metabolism (ko00473), ether lipid metabolism (ko00565), glycerolipid metabolism (ko00561), glycolysis/gluconeogenesis (ko00010). Ascorbate and aldarate metabolism (ko00053) and isoquinoline alkaloid biosynthesis (ko00950) were significantly changed for the “species” variable (BH, *p* < 0.05). 

### 3.6. Quantitative Analysis of 16S rRNA Gene 

**Figure 7:**
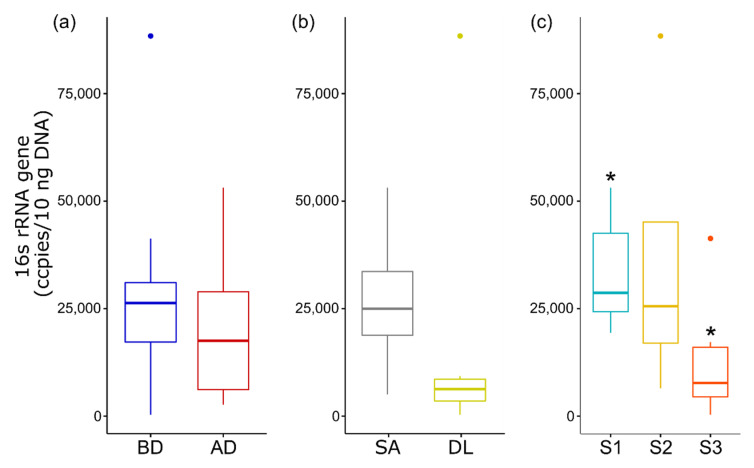
The total bacterial load of eggs from the three aquaculture sites quantified by qPCR of 16S rRNA. The box plots represent the different variables: (**a**) disinfection—before disinfection (BD) and after disinfection (AD); (**b**) species—gilthead seabream (SA) and European seabass (DL); (**c**) site—site 1–3. * Significance code: at *p* < 0.05.

The total bacterial load of the eggs before and after disinfection, based on the quantification of the 16S rRNA gene, did not change significantly (Figure 7). However, the average load of total bacteria decreased after eggs’ disinfection. No significant difference was detected in the bacterial load of eggs from different species, while the bacterial load of the seabass eggs was lower than that of the seabream eggs. A significant difference was observed in the total bacterial load of the eggs collected from site 1 compared to site 3 (Figure 7). 

## 4. Discussion

Metagenomic studies of European seabass and gilthead seabream egg microbiomes using next-generation sequencing have not been reported, despite the economic value of the species and the risks to production of diseases during the hatchery stage [27]. The present study carried out metagenomic profiling to identify the bacterial communities associated with eggs and water from commercial hatcheries of European seabass and gilthead seabream. 

The results of our study and previous studies on channel catfish eggs [24] and gilthead seabream larvae at 2 and 34 days post-hatch (dph) [40] suggest that Proteobacteria and Bacteroidetes are the most abundant and probably most common phyla colonizing fish eggs and larval fish stages. However, our data revealed that the relative abundance of the main bacterial phyla varied with site, as shown by the high relative abundance of Cyanobacteria and relatively less abundant Firmicutes in most of the samples from site 3 and in some of the samples from site 2, compared to site 1. High relative abundance of Cyanobacteria was previously reported in the microbiota of eggs and larvae of the channel catfish [41] and in juveniles of grass carp (*Ctenopharyngodon idella*) [41], both freshwater species. Since the broodstock water in sites 2 and 3 had a lower temperature (mean ≅ 16 °C) and salinity than site 1, environmental conditions including these two factors may explain the higher abundance of Cyanobacteria in these sites. Studies characterizing the growth and physiology of Cyanobacteria have demonstrated that temperature and salinity directly influence their growth [42]. Corroborating evidence for the importance of salinity on fish-associated microbiota also comes from studies of the skin-associated microbiota in Atlantic salmon (*Salmo salar*) transitioning between fresh and saltwater. Firmicutes, Actinobacteria, Verrucomicrobia, and Cyanobacteria were more abundant in Atlantic salmon skin microbiota in freshwater compared to the skin microbiota in seawater, indicating that their abundance was highly affected by salinity [43].

Most of the bacterial families with high relative abundance in the present study (Vibrionaceae, Colwelliaceae, Pseudoalteromonadaceae, Alteromonadaceae, Shewanellaceae, Saccharospirillaceae, and Thiotrichaceae) were also found to be among the top egg bacterial families in ballan wrasse at a commercial marine hatchery [25]. Another abundant family, Flavobacteriaceae, with 17.6% average abundance in water and 6.8% in the egg samples, was also one of the most abundant families of bacteria in fertilized brown trout eggs but not in the ballan wrasse egg microbiota [25,44]. This suggests that Flavobacteriaceae (Flavobacteriales order) may be an example of a site-specific bacterial family. Cryomorphaceae (another family of the Flavobacteriales order) and Rhodobacteraceae were among the most abundant families detected in the broodstock water of gilthead seabream from site 1. Interestingly, Cryomorphaceae was also highly abundant in the tank water of 34 dph gilthead seabream larvae compared to the tank water of 2 dph larvae, and Rhodobacteraceae was abundant at all stages in the tank water and in the food source (*Artemia nauplii*) [40]. Rhodobacteraceae were among the most abundant bacterial families in lumpfish (*Cyclopterus lumpus* L.) rearing water and eggs [45]. Taken together, the results of our study and previous studies [40,45] highlight the likely importance of Rhodobacteraceae in marine fish egg microbiota and the relative and possibly significant contribution of microbiota present in water and food. 

In our study, Thiohalorhabdaceae and Arcobacteraceae showed higher relative abundance on eggs collected from some of the broodstock than in the corresponding broodstock water. Comparing the results of the fish egg microbiota in the present and previous studies [25,44,45,46] reveals that Thiohalorhabdaceae and Arcobacteraceae were uncommon and not always present in fish egg microbiota. Geographical location/local conditions may account for the presence of these bacterial families in eggs from gilthead seabream and European seabass. Support for this idea comes from studies showing that algal samples collected from the French Tamaris coast situated in the Mediterranean sea were highly abundant in Thiohalorhabdaceae [47]. Broodstock genetics and health status have been proposed to influence the egg microbial community of brown trout [46] and the gut and skin microbiota of the Atlantic Salmon (*Salmo salar*) [48]. It remains to be established if the higher relative abundance of some bacterial families (e.g., Thiohalorhabdaceae) in the egg microbiota in our study using broodstock from different companies and species was not only driven by the surrounding water but also broodstock genetics. 

Direct and indirect factors that can cause pathogenic bacteria to spread in hatcheries include broodstock health, diet, water, and vertical transmission from gonadal fluids [49,50,51]. Therefore, providing an extremely clean environment is one of the key challenges in hatcheries and, for this reason, egg disinfection postfertilization is a common practice, with the aim of eliminating opportunistic diseases [52]. A range of disinfectants and protocols based on hydrogen peroxide, glutaraldehyde, ozone, and iodophors are available for the treatment of eggs to decrease bacterial/fungal loads, and their effectiveness depends on factors such as pH and temperature [52]. Analysis of the impact of the iodine-based disinfectant protocols on the egg microbiota in the present study suggested that they were largely ineffective and did not significantly impact the alpha- or beta-diversity, the relative abundance, or the total bacterial load of the bacterial community of seabass and seabream eggs. The results of previous studies using conventional microbiological approaches are contradictory in relation to the effectiveness of iodine-based disinfectants on specific pathogenic bacteria of the egg surface, e.g., ranging from total removal to the absence of an effect of iodophors on *Flavobacterium psychrophilum* [52], and total bacterial loads in cultures of egg bacterial communities, including *Vibrio* spp. [53]. Two factors are proposed to explain the poor disinfection capacity of iodine-based treatments: (i) the majority of bacteria are inside the eggs or strongly attached to the egg chorion and so superficial disinfection protocols have no effect, and/or (ii) the current protocols for iodine-based disinfectants are inadequate and optimization of disinfection protocols is required. An important caveat of most microbiome studies, including the present study, is that 16S rRNA can be amplified from both viable and dead bacteria. Disinfectants such as iodine rapidly penetrate microorganisms and attack key groups of proteins [54] and it is possible that, because the egg samples were taken immediately after treatment, DNA from dead bacteria still had not broken down. Further studies are needed that couple metagenomics and the assessment of bacterial viability to provide a better understanding of the efficacy of iodine-based disinfectants.

The egg and water microbial communities had significantly different beta-diversity and abundance, and included 36 out of 2444 significantly different OTUs between the two sample types. This suggests that a relatively small number of bacteria may explain the separation between the microbiota of broodstock water and eggs observed in the PcoA analysis. However, with the higher relative abundance of some potentially pathogenic bacteria in gilthead seabream and European seabass eggs, such as *Pseudomonas*, *Pseudoalteromonas*, *Leucothrix*, and *Arcobacter*, more studies are needed to understand their growth dynamics and pathogenicity to establish risk. The presence in production systems of pathogenic bacteria such as some *Vibrio* and *Photobacterium* species was reported to cause mass mortality of gilthead seabream and European seabass larvae and juveniles [55]. Bacterial pathogens of adult fish such as *Pseudomonas* and *Flavobacterium psychrophilum*, which cause bacterial cold-water disease (BCWD), have previously been detected at the egg stage of brown trout (*Salmo trutta*) [44]. Furthermore, mass mortalities of cod (*Gadus morhua*) eggs identified, as the causal factor, a pathogenic bacterial species, *Leucothrix mucor* [56]. Although we did not detect large variation in the microbial community abundance between European seabass and gilthead seabream eggs, the relatively higher abundance of two *Pseudomonas* OTUs and one *Photobacterium* OTU in the European seabass eggs were examples of species-specific OTUs. Therefore, the results of the present study support the idea proposed from observations of cod and halibut eggs in the 1980s that the ability of bacteria to colonize eggs may depend on the nature of the egg chorion or presence of bactericidal enzymes (e.g., lysozyme and lectins) [57]. It will be important in the future to study the functional role of the egg microbiota and to establish which are beneficial and which are pathogenic members of the microbial community. The use of metagenomics and complimentary approaches such as PCR, quantitative PCR, and bacterial culture will extend our understanding of the contribution of the microbial community to fish egg physiology and quality [58,59].

The 16S rRNA gene qPCR analysis and the functional inference analysis further supported the site/geographical location’s effect on the egg microbial community and suggested that “site” was the main factor determining the egg microbial community compared to all the other factors analyzed. The association of some pathways such as caprolactam degradation, geraniol degradation, and benzoate degradation with disinfection suggests that, in addition to the elimination of bacteria, they may favor the maintenance of some bacterial genera or species. The reported antibacterial effects on some bacterial genera of benzoate [60], geraniol [61,62], and caprolactam and the degradation of these compounds by others further support the idea of alternative secondary effects for disinfectants [63,64,65]. 

**Figure 8:**
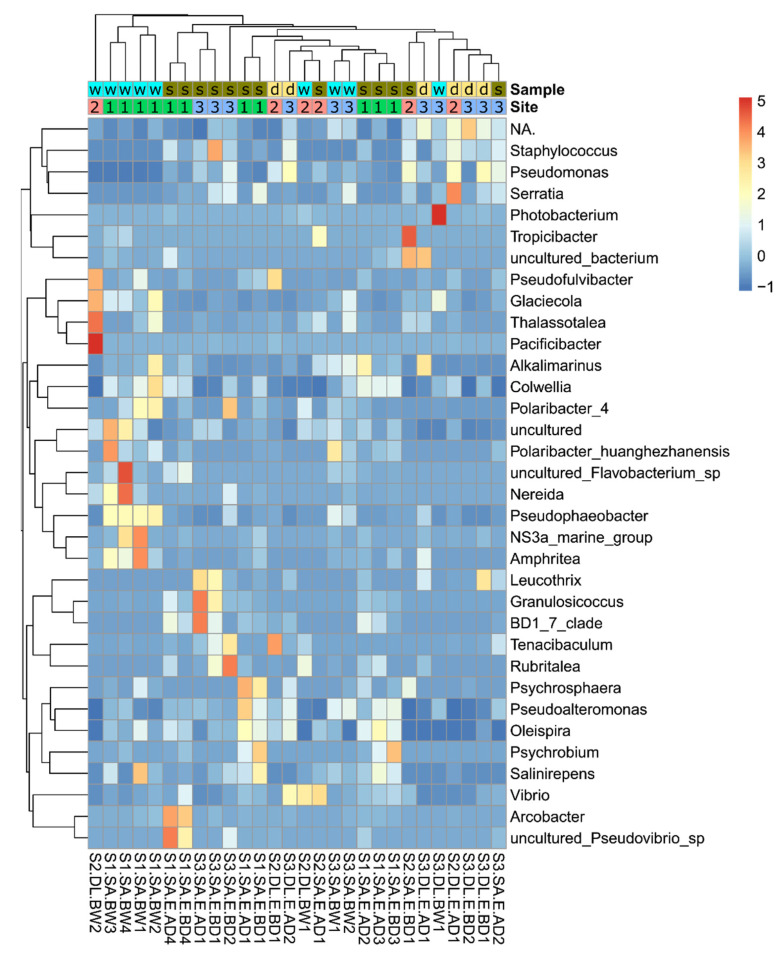
The heatmap of top bacterial genera across eggs and broodstock holding water. Sample: gilthead seabream (s), European seabass (d), water (w); site: site 1–3.

The most abundant bacterial genera were highlighted across all eggs and water samples (Figure 8). In general, an association of the microbiome with the site, species, and type of sample (water/egg) was observed. The contribution of the broodstock water to the egg microbiome was evident from the detection of a similar bacterial composition in eggs and water. The bacterial genera with significant changes in their relative abundance were also identified based on variables such as site, species, and type of sample (eggs and water). Optimized methods for sample collection and processing were developed, and the usefulness of current disinfection protocols for eggs collected from broodstock water was determined. The similarity and differences found between gilthead seabream and European seabass egg microbiomes were identified. The contribution of different factors (species, site, and disinfection) to the bacterial community of eggs and broodstock water in commercial hatcheries was established and this knowledge will contribute to the development of future strategies for hatchery management.

In summary, the results show that Proteobacteria are the preponderant phyla of the bacterial community found in both European seabass and gilthead seabream eggs (before or after disinfection) and also in broodstock water across different hatcheries. Vibrio sp. was the most highly represented genus (7.1% on average), followed by Glaciecola (4.8%), Pseudoalteromonas (4.4%), and Colwellia (4.2%), in eggs and water across the sites. Site, species, and type of sample (eggs or water) drove the microbial community structure and influenced microbiome functional profiles. The overall composition and the relative abundance of bacterial genera of the eggs and water microbiome were similar, but differences were found in the relative abundance of some bacterial genera/OTUs. This indicates that the water microbiome makes a high contribution to the eggs’ bacterial communities. The results of our study highlight the need for further investigation into the egg microbiome and the importance for hatcheries of optimized disinfection protocols that take into consideration the initial bacterial composition, disinfectant composition, and the species. The risk of some potential pathogenic species related to the Pseudomonas, Pseudoalteromonas, Leucothrix, and Arcobacter genera, with high relative abundance in egg samples compared to water, needs to be assessed in future studies.

## Figures and Tables

**Table 1:** Broodstock characteristics and conditions (linked to Figure 1).

Site	BS	Species	Tank	Male (*n*)	Female (*n*)	Density (kg/m^3^)	Weight ^֎^ (kg)
1	BS 1	SA	1	10	31	8.36	2.02
1	BS 2	SA	2	11	20	5.28	2.21
1	BS 3	SA	3	16	30	5.82	2.23
1	BS 4	SA	4	10	27	6.78	3.01
2	BS 1	SA	1	38	105	5.9	1.90
2	BS 1	DL	1	67	68	14.9	5.00
2	BS 2	DL	2	50	75	7.7	2.80
3	BS 1	SA	1	63	20	5.29	1.59
3	BS 2	SA	2	23	25	3.45	1.79
3	BS 1	DL	1	23	28	7.11	3.48
3	BS 2	DL	2	25	31	8.61	3.81

BS = Broodstock; *n* = number of fish; ֎ = weight expressed as the mean/tank.

**Table 2:** PERMANOVA analysis (nr. of permutations = 1000) across all egg and water samples based on two factors: aquaculture site (Site 1, Site 2, and Site 3) and type of sample (egg vs. water).

	Df	Sum Sq	Mean Sq	F. Model	R2	Pr (>F)
Site	1	1.2367	1.23667	3.9341	0.12130	0.000999 ***
Sample type (eggs vs. water)	1	1.0998	1.09975	3.4986	0.10787	0.000999 ***
Residuals	25	7.8586	0.31434		0.77083	
Total	27	10.1950			1.00000	

Df: degrees of freedom; Sq: square; Significant code: *** *p* < 0.001.

**Table 3:** PERMANOVA analysis (permutation = 1000) across egg samples based on three factors: aquaculture site (Site 1, Site 2, and Site 3), species (European seabass vs. gilthead seabream eggs), and disinfection (egg before vs. after disinfection).

	Df	Sum Sq	Mean Sq	F. Model	R2	Pr (>F)
Site	1	1.0757	1.07570	3.6486	0.16206	0.000999 ***
Species	1	0.7011	0.70111	2.3780	0.10563	0.000999 ***
Disinfection	1	0.1703	0.17031	0.5777	0.02566	0.962038
Site: Species	1	0.5628	0.56280	1.9089	0.08479	0.005994 **
Residuals	14	4.1276	0.29483		0.62186	
Total	18	6.6375			1.00000	

Df: degrees of freedom; Sq: square; Significant code: *** *p* < 0.001, ** *p* < 0.01.

**Table 4:** The means of the relative abundance (%) of the top 10 pathways across the different variables.

Pathway		Site			Species		Type		Disinfection		All
KO	Name	S1	S2	S3	Sa	Dl	E	W	B	A	
ko01230	Biosynthesis of amino acids	6.7	7.4	7.2	6.9	7.4	7.0	7.1	7.0	7.1	7.0
ko01200	Carbon metabolism	6.9	6.7	6.6	6.7	6.6	6.7	6.8	6.7	6.7	6.7
ko00230	Purine metabolism	4.3	4.7	4.5	4.3	4.5	4.4	4.6	4.5	4.3	4.5
ko00240	Pyrimidine metabolism	3.1	3.3	3.3	3.1	3.4	3.2	3.4	3.3	3.1	3.2
ko00620	Pyruvate metabolism	2.9	2.9	2.9	2.9	2.9	2.9	3.0	2.9	2.9	2.9
ko00330	Arginine and proline metabolism	2.4	2.5	2.5	2.4	2.5	2.4	2.4	2.4	2.4	2.4
ko00260	Glycine, serine, and threonine metabolism	2.3	2.3	2.4	2.3	2.3	2.3	2.5	2.3	2.2	2.3
ko00250	Alanine, aspartate, and glutamate metabolism	2.3	2.3	2.3	2.3	2.2	2.3	2.3	2.3	2.3	2.3
ko00010	Glycolysis/Gluconeogenesis	2.1	2.2	2.4	2.1	2.4	2.2	2.3	2.2	2.2	2.2
ko00720	Carbon fixation pathways in prokaryotes	2.2	2.3	2.2	2.2	2.3	2.2	2.3	2.2	2.2	2.2

S1 = site 1, S2 = site 2, S3 = site 3; Sa = gilthead seabream, Dl = European seabass; E = egg, W = water; B = before, A = after.

## Data Availability

The metagenomics raw data generated during this study were deposited at NCBI SRA (sequence read archive) under project number PRJNA727018 (under embargo until the publication is released).

## Supplementary Materials

The following are available online at https://www.mdpi.com/article/10.3390/microorganisms9112275/s1, Table S1. Sequencing statistics of the 28 libraries prepared from European seabass and gilthead seabream eggs before and after disinfection and from broodstock water. More information about the sample labels is provided in Figure 1. Table S2. Bacterial composition and their relative abundance in each of the samples. A total of 294 OTUs were selected, using as the criteria their presence at >1% relative abundance in the 28 samples studied. NA means that the OTU at the taxonomic level was not identified through comparison with the SILVA database. Table S3. The top 15 genera detected in each egg and water sample. The relative percentage of each genus was calculated in relation to all detected genera in each sample. Information on sample labels is provided in Figure 1. Table S4. Differential abundance analysis of microbiota of eggs and water using three factors: Site (Site 1, Site 2, Site 3), type of sample (egg vs. water), and species (European seabass vs. gilthead seabream). OTUs with significantly differential (relative) abundance are presented in different Excel sheets in the table for site, broodstock water, egg, and species. The R package ALDEx2 (1.20.0) was run to identify the OTUs that were differentially abundant across the conditions tested. The centered log-ratio-transformed values were analyzed with a general linear model (glm) and a Kruskal–Wallis test applied. The ALDEx2 output included the expected *p*-value of a Kruskal–Wallis test (kw.ep), expected Benjamini–Hochberg-corrected *p*-value of a Kruskal–Wallis test (kw.eBH), expected *p*-value of a glm test (glm.ep), and expected Benjamini–Hochberg-corrected *p*-value of a glm test (glm.eBH). Table S5. KEGG ontology terms and association of the pathways with the site (Site 1, Site 2, Site 3), disinfection usage, and species (European seabass and gilthead seabream). Functional prediction was performed using Tax4Fun and identified 6311 KEGG Orthology terms and 133 pathways. Functional predictions of the metagenomics profiles were performed using the web-based platform Microbiome Analyst and Tax4Fun. The associations between functional categories and the experimental factor (site, species, disinfection) were tested based on the global test algorithm and are presented in different Excel sheets in the folder. Statistic.Q and Expected.Q = test for homogeneity; Pval = *p*-value; Holm.p = *p*-value for Holm–Bonferroni method; FDR = false discovery rate.

## References

[1] Gilbert S.F., Sapp J., Tauber A.I. (2012). A symbiotic view of life: We have never been individuals. Q. Rev. Biol..

[2] McFall-Ngai M., Hadfield M.G., Bosch T.C.G., Carey H.V., Domazet-Lošo T., Douglas A.E., Dubilier N., Eberl G., Fukami T., Gilbert S.F. (2013). Animals in a bacterial world, a new imperative for the life sciences. Proc. Natl. Acad. Sci. USA.

[3] Pasolli E., Asnicar F., Manara S., Zolfo M., Karcher N., Armanini F., Beghini F., Manghi P., Tett A., Ghensi P. (2019). Extensive unexplored human microbiome diversity revealed by over 150,000 genomes from metagenomes spanning age, geography, and lifestyle. Cell.

[4] Proctor L.M., Creasy H.H., Fettweis J.M., Lloyd-Price J., Mahurkar A., Zhou W., Buck G.A., Snyder M.P., Strauss J.F., Weinstock G.M. (2019). The Integrative human microbiome project. Nature.

[5] Hadrich D. (2018). Microbiome research is becoming the key to better understanding health and nutrition. Front. Genet..

[6] Brugman S., Ikeda-Ohtsubo W., Braber S., Folkerts G., Pieterse C.M.J., Bakker P.A.H.M. (2018). A Comparative review on microbiota manipulation: Lessons from fish, plants, livestock, and human research. Front. Nutr..

[7] Pickard J.M., Zeng M.Y., Caruso R., Núñez G. (2017). Gut microbiota: Role in pathogen colonization, immune responses, and inflammatory disease. Immunol. Rev..

[8] Simon C., Daniel R. (2011). Metagenomic analyses: Past and future trends. Appl. Environ. Microbiol..

[9] Aßhauer K.P., Wemheuer B., Daniel R., Meinicke P. (2015). Tax4Fun: Predicting functional profiles from metagenomic 16S rRNA data. Bioinformatics.

[10] Groff J.M. (2001). Cutaneous biology and diseases of fish. Vet. Clin. North Am. Exot. Anim. Pract..

[11] Llewellyn M.S., Boutin S., Hoseinifar S.H., Derome N. (2014). Teleost microbiomes: The state of the art in their characterization, manipulation and importance in aquaculture and fisheries. Front. Microbiol..

[12] Egerton S., Culloty S., Whooley J., Stanton C., Ross R.P. (2018). The gut microbiota of marine fish. Front. Microbiol..

[13] Legrand T.P.R.A., Catalano S.R., Wos-Oxley M.L., Stephens F., Landos M., Bansemer M.S., Stone D.A.J., Qin J.G., Oxley A.P.A. (2018). The inner workings of the outer surface: Skin and gill microbiota as indicators of changing gut health in yellowtail kingfish. Front. Microbiol..

[14] Legrand T.P.R.A., Wynne J.W., Weyrich L.S., Oxley A.P.A. (2020). A microbial sea of possibilities: Current knowledge and prospects for an improved understanding of the fish microbiome. Rev. Aquac..

[15] Tellez G., Higgins S.E., Donoghue A.M., Hargis B.M. (2006). Digestive physiology and the role of microorganisms. J. Appl. Poult. Res..

[16] Newaj-Fyzul A., Al-Harbi A.H., Austin B. (2014). Review: Developments in the use of probiotics for disease control in aquaculture. Aquaculture.

[17] Ringø E., Dimitroglou A., Hoseinifar S.H., Davies S.J., Merrifield D.L., RingØ E. (2014). Prebiotics in finfish: An update. Aquaculture Nutrition: Gut Health, Probiotics and Prebiotics.

[18] Wang A.R., Ran C., Ringø E., Zhou Z.G. (2018). Progress in fish gastrointestinal microbiota research. Rev. Aquac..

[19] Hansen G.H., Olafsen J.A. (1999). Bacterial interactions in early life stages of marine cold water fish. Microb. Ecol..

[20] Olafsen J.A. (2001). Interactions between fish larvae and bacteria in marine aquaculture. Aquaculture.

[21] Merrifield D.L., Rodiles A., Beck B.H., Peatman E. (2015). The fish microbiome and its interactions with mucosal tissues. Mucosal Health Aquaculture.

[22] Borges N., Keller-Costa T., Sanches-Fernandes G.M.M., Louvado A., Gomes N.C.M., Costa R. (2021). Bacteriome structure, function, and probiotics in fish larviculture: The good, the bad, and the gaps. Annu. Rev. Anim. Biosci..

[23] Vadstein O., Bergh Ø., Gatesoupe F.-J., Galindo-Villegas J., Mulero V., Picchietti S., Scapigliati G., Makridis P., Olsen Y., Dierckens K. (2013). Microbiology and immunology of fish larvae. Rev. Aquac..

[24] Abdul Razak S., Griffin M.J., Mischke C.C., Bosworth B.G., Waldbieser G.C., Wise D.J., Marsh T.L., Scribner K.T. (2019). Biotic and abiotic factors influencing channel catfish egg and gut microbiome dynamics during early life stages. Aquaculture.

[25] Bone A., Bekaert M., Papadopoulou A., McMillan S., Adams A., Davie A., Desbois A.P. (2020). Bacterial communities of ballan wrasse (*Labrus bergylta*) eggs at a commercial marine hatchery. Curr. Microbiol..

[26] FEAP Federation of European Aquaculture Producers Annual Report. https://feap.info/index.php/data/.

[27] Muniesa A., Basurco B., Aguilera C., Furones D., Reverté C., Sanjuan-Vilaplana A., Jansen M.D., Brun E., Tavornpanich S. (2020). Mapping the knowledge of the main diseases affecting sea bass and sea bream in Mediterranean. Transbound. Emerg. Dis..

[28] Yoder M., De Ley I.T., King I.W., Mundo-Ocampo M., Mann J., Blaxter M., Poiras L., Ley P. (2006). De DESS: A versatile solution for preserving morphology and extractable DNA of nematodes. Nematology.

[29] Pinto P.I.S., Guerreiro C.C., Costa R.A., Martinez-Blanch J.F., Carballo C., Codoñer F.M., Manchado M., Power D.M. (2019). Understanding pseudo-albinism in sole (*Solea senegalensis*): A transcriptomics and metagenomics approach. Sci. Rep..

[30] Klindworth A., Pruesse E., Schweer T., Peplies J., Quast C., Horn M., Glöckner F.O. (2013). Evaluation of general 16S ribosomal RNA gene PCR primers for classical and next-generation sequencing-based diversity studies. Nucleic Acids Res..

[31] Andrews S. Babraham Bioinformatics—FastQC a Quality Control Tool for High Throughput Sequence Data. http://www.bioinformatics.babraham.ac.uk/projects/fastqc.

[32] Caporaso J.G., Kuczynski J., Stombaugh J., Bittinger K., Bushman F.D., Costello E.K., Fierer N., Pẽa A.G., Goodrich J.K., Gordon J.I. (2010). QIIME allows analysis of high-throughput community sequencing data. Nat. Methods.

[33] Callahan B.J., McMurdie P.J., Rosen M.J., Han A.W., Johnson A.J.A., Holmes S.P. (2016). DADA2: High-resolution sample inference from Illumina amplicon data. Nat. Methods.

[34] Quast C., Pruesse E., Yilmaz P., Gerken J., Schweer T., Yarza P., Peplies J., Glöckner F.O. (2013). The SILVA ribosomal RNA gene database project: Improved data processing and web-based tools. Nucleic Acids Res..

[35] Chong J., Liu P., Zhou G., Xia J. (2020). Using Microbiome Analyst for comprehensive statistical, functional, and meta-analysis of microbiome data. Nat. Protoc..

[36] Goeman J.J., Van de Geer S., De Kort F., van Houwellingen H.C. (2004). A global test for groups of genes: Testing association with a clinical outcome. Bioinformatics.

[37] Caporaso J.G., Lauber C.L., Walters W.A., Berg-Lyons D., Huntley J., Fierer N., Owens S.M., Betley J., Fraser L., Bauer M. (2012). Ultra-high-throughput microbial community analysis on the Illumina HiSeq and MiSeq platforms. ISME J..

[38] Apprill A., McNally S., Parsons R., Weber L. (2015). Minor revision to V4 region SSU rRNA 806R gene primer greatly increases detection of SAR11 bacterioplankton. Aquat. Microb. Ecol..

[39] Parada A.E., Needham D.M., Fuhrman J.A. (2016). Every base matters: Assessing small subunit rRNA primers for marine microbiomes with mock communities, time series and global field samples. Environ. Microbiol..

[40] Califano G., Castanho S., Soares F., Ribeiro L., Cox C.J., Mata L., Costa R. (2017). Molecular taxonomic profiling of bacterial communities in a Gilthead Seabream (*Sparus aurata*) hatchery. Front. Microbiol..

[41] Zeng A., Tan K., Gong P., Lei P., Guo Z., Wang S., Gao S., Zhou Y., Shu Y., Zhou X. (2020). Correlation of microbiota in the gut of fish species and water. 3 Biotech.

[42] Silveira S.B., Odebrecht C. (2019). Effects of salinity and temperature on the growth, toxin production, and akinete germination of the Cyanobacterium *Nodularia Spumigena*. Front. Mar. Sci..

[43] Lokesh J., Kiron V. (2016). Transition from freshwater to seawater reshapes the skin-associated microbiota of Atlantic salmon. Sci. Rep..

[44] Wilkins L.G.E., Rogivue A., Schütz F., Fumagalli L., Wedekind C. (2015). Increased diversity of egg-associated bacteria on brown trout (*Salmo trutta*) at elevated temperatures. Sci. Rep..

[45] Roalkvam I., Drønen K., Dahle H., Wergeland H.I. (2019). Microbial communities in a flow-through fish farm for lumpfish (*Cyclopterus lumpus* L.) during healthy rearing conditions. Front. Microbiol..

[46] Wilkins L.G.E., Fumagalli L., Wedekind C. (2016). Effects of host genetics and environment on egg-associated microbiotas in brown trout (*Salmo trutta*). Mol. Ecol..

[47] Paix B., Carriot N., Barry-Martinet R., Greff S., Misson B., Briand J.F., Culioli G. (2020). A multi-omics analysis suggests links between the differentiated surface metabolome and epiphytic microbiota along the thallus of a Mediterranean Seaweed holobiont. Front. Microbiol..

[48] Uren Webster T.M., Consuegra S., Hitchings M., Garcia de Leaniz C. (2018). Interpopulation variation in the Atlantic salmon microbiome reflects environmental and genetic diversity. Appl. Environ. Microbiol..

[49] Migaud H., Bell G., Cabrita E., Mcandrew B., Davie A., Bobe J., Herráez M.P., Carrillo M. (2013). Gamete quality and broodstock management in temperate fish. Rev. Aquac..

[50] Van Vliet D., Loch T.P., Faisal M. (2015). Flavobacterium psychrophilum infections in salmonid broodstock and hatchery-propagated stocks of the Great Lakes basin. J. Aquat. Anim. Health.

[51] Pradeep P.J., Suebsing R., Sirthammajak S., Kampeera J., Jitrakorn S., Saksmerprome V., Turner W., Palang I., Vanichviriyakit R., Senapin S. (2016). Evidence of vertical transmission and tissue tropism of Streptococcosis from naturally infected red tilapia (*Oreochromis spp.*). Aquac. Reports.

[52] De Swaef E., Van den Broeck W., Dierckens K., Decostere A. (2016). Disinfection of teleost eggs: A review. Rev. Aquac..

[53] Can E., Saka Ş., Firat M.K. (2010). Disinfection of gilthead sea bream (*Sparus aurata*), red porgy (*Pagrus pagrus*), and common Dentex (*Dentex dentex*) eggs from Sparidae with different disinfectants. Kafkas Üniversitesi Vet. Fakültesi Derg..

[54] McDonnell G., Russell A.D. (1999). Antiseptics and disinfectants: Activity, action, and resistance. Clin. Microbiol. Rev..

[55] Abdel-Aziz M., Eissa A.E., Hanna M., Okada M.A. (2013). Identifying some pathogenic *Vibrio*/*Photobacterium* species during mass mortalities of cultured Gilthead seabream (*Sparus aurata*) and European seabass (*Dicentrarchus labrax*) from some Egyptian coastal provinces. Int. J. Vet. Sci. Med..

[56] Johnson P.W., Sieburth J.M., Sastry A., Arnold C.R., Doty M.S. (1971). *Leucothrix mucor* infestation of benthic crustacea, fish eggs, and tropical algae. Limnol. Oceanogr..

[57] Hansen G.H., Olafsen J.A. (1989). Bacterial colonization of cod (*Gadus morhua* L.) and halibut (*Hippoglossus hippoglossus*) eggs in marine aquaculture. Appl. Environ. Microbiol..

[58] Fusco V., Quero G.M., Ayman A.E. (2012). Nucleic acid-based methods to identify, detect and type pathogenic bacteria occurring in milk and dairy products. Structure and Function of Food Engineering.

[59] Lazcka O., Del Campo F.J., Munoz F.X. (2007). Pathogen detection: A perspective of traditional methods and biosensors. Biosens. Bioelectron..

[60] Metwally N.H., Mohamed M.S. (2020). New imidazolone derivatives comprising a benzoate or sulfonamide moiety as anti-inflammatory and antibacterial inhibitors: Design, synthesis, selective COX-2, DHFR and molecular-modeling study. Bioorg. Chem..

[61] Kim J.M., Marshall M.R., Cornell J.A., Iii J.P., Wei C.I. (1995). Antibacterial activity of carvacrol, citral, and geraniol against *Salmonella typhimurium* in culture medium and on fish cubes. J. Food Sci..

[62] Pontes E.K.U., Melo H.M., Nogueira J.W.A., Firmino N.C.S., de Carvalho M.G., Catunda Júnior F.E.A., Cavalcante T.T.A. (2019). Antibiofilm activity of the essential oil of citronella (*Cymbopogon nardus*) and its major component, geraniol, on the bacterial biofilms of *Staphylococcus aureus*. Food Sci. Biotechnol..

[63] Höschle B., Jendrossek D. (2005). Utilization of geraniol is dependent on molybdenum in *Pseudomonas aeruginosa*: Evidence for different metabolic routes for oxidation of geraniol and citronellol. Microbiology.

[64] Uhlik O., Wald J., Strejcek M., Musilova L., Ridl J., Hroudova M., Vlcek C., Cardenas E., Mackova M., Macek T. (2012). Identification of bacteria utilizing biphenyl, benzoate, and naphthalene in long-term contaminated soil. PLoS ONE.

[65] Baxi N.N. (2013). Influence of ε-caprolactam on growth and physiology of environmental bacteria. Ann. Microbiol..

